# Incisional Hernia at Extraction Sites Following Laparoscopic Colorectal Resection: A Comparative Study of Midline vs Non-midline Extraction

**DOI:** 10.7759/cureus.96403

**Published:** 2025-11-09

**Authors:** Naveenkumar Viswanathan, Felix Nicholas, Shreeshail Dayanand, Sonali Manoharan

**Affiliations:** 1 General Surgery, Prince Charles Hospital, Merthyr Tydfil, GBR; 2 General Surgery, Prince Charles Hospital, Merthyr Tydfi, GBR; 3 Paediatrics, Prince Charles Hospital, Merthyr Tydfil, GBR

**Keywords:** colorectal surgery, extraction site, incisional hernia, laparoscopy, surgical wound infection

## Abstract

Introduction: The choice of specimen extraction site in laparoscopic colorectal surgery may influence incisional hernia risk, yet optimal site selection remains debated. This study compares hernia rates between midline and non-midline extraction sites, assessing the impact of surgical site infection (SSI), diabetes, and immunosuppression.

Methods: A retrospective cohort study included 276 patients undergoing laparoscopic colorectal cancer resection (2019-2022) at a UK District General Hospital, with a minimum three-year follow-up. Patients were grouped by extraction site: midline (n=99) and non-midline (n=177; transverse off-midline, n=116; Pfannenstiel, n=37; stoma site, n=24). Data on age, BMI, American Society of Anesthesiologists (ASA) grade, diabetes, immunosuppression, surgical site infection (SSI), and incisional hernia (diagnosed via CT or clinical examination) were collected. Statistical analyses included Chi-square, Fisher’s exact, t-tests, and Pearson correlations.

Results: Midline extractions had a higher hernia rate (27.3%) than non-midline (11.9%; p=0.0021). Pfannenstiel incisions showed the lowest rate (2.8%; p=0.0008), followed by transverse (14.7%; p=0.0343), with stoma site rates similar to midline (12.5%; p=0.1859). SSI strongly correlated with hernia incidence (r=0.950, p=0.013), unlike diabetes (r=0.296, p=0.628) or immunosuppression (r=0.284, p=0.640). ASA grade distribution differed significantly (p=0.0324).

Conclusion: Non-midline extraction sites, particularly Pfannenstiel, reduce incisional hernia risk compared to midline incisions. SSI is a critical risk factor, emphasizing the importance of infection prevention. These findings support strategic site selection to optimize outcomes.

## Introduction

Laparoscopic colorectal surgery has become a cornerstone of modern surgical practice, offering reduced postoperative pain, shorter hospital stays, and improved cosmetic outcomes compared to open approaches [1,2]. Despite these advantages, postoperative complications, such as incisional hernias, remain a significant concern, affecting patient quality of life and increasing healthcare costs [3,4]. Incisional hernias, characterized by fascial defects at surgical incision sites, occur in 20-30% of patients undergoing abdominal surgery, with rates varying by incision type and patient factors [5,6]. In laparoscopic colorectal surgery, the specimen extraction site is a critical determinant of hernia risk, as it requires a larger incision than trocar sites, compromising abdominal wall integrity [7,8].

The choice of extraction site-midline, transverse off-midline, Pfannenstiel, or stoma site-may influence hernia incidence due to differences in anatomical stress distribution and wound healing dynamics [9,10]. Midline incisions, commonly used for their accessibility, are associated with higher hernia rates due to increased tension and weaker fascial layers [11,12]. In contrast, non-midline incisions, such as Pfannenstiel, benefit from lower tension and muscle support, potentially reducing hernia risk [2,3]. Surgical site infection (SSI), a known risk factor for hernia formation, further complicates outcomes, with studies reporting odds ratios as high as 3.2 for hernia development in infected wounds [4,13]. Patient-related factors, including diabetes and immunosuppression, have also been implicated, though their impact remains inconsistent across studies [7,14].

Despite growing evidence, the optimal extraction site to minimize incisional hernia risk in laparoscopic colorectal surgery remains unclear, particularly in the context of SSI and patient comorbidities [9,10]. This study aims to compare incisional hernia rates between midline and non-midline extraction sites (transverse off-midline, Pfannenstiel, and stoma site) in patients undergoing laparoscopic colorectal cancer resection, while assessing the influence of SSI, diabetes, and immunosuppressant use. The primary objective of this study was to compare the incidence of incisional hernia between midline and non-midline extraction sites following laparoscopic colorectal resection. The secondary objectives were to evaluate the associations between surgical site infection, diabetes, and immunosuppression and the development of incisional hernia.

## Materials and methods

This retrospective cohort study included patients who underwent laparoscopic colorectal cancer resection between March 2019 and March 2022 at a UK District General Hospital. Patients were eligible if they had a minimum of three years of follow-up, confirmed by clinical records or imaging. Exclusion criteria comprised conversion to open surgery, insufficient follow-up (less than three years), or incomplete medical records. This study was reviewed using the National Health Service (NHS) Health Research Authority (HRA) decision tool for Wales, which confirmed that NHS Research Ethics Committee (REC) review was not required for this project. All other relevant institutional approvals were obtained prior to commencement of the study. The study used anonymised retrospective data and did not require individual consent. All data handling complied with General Data Protection Regulation (GDPR) and institutional protocols, in line with the Declaration of Helsinki.

Data collection: Patients were grouped by specimen extraction site into midline and non-midline cohorts, with the latter subdivided into transverse off-midline, Pfannenstiel, and stoma site incisions. Data were extracted from electronic medical records and included. For right-sided colectomies, extracorporeal anastomosis was typically performed using a periumbilical or midline incision for specimen extraction and anvil placement. In left-sided and sigmoid resections, Pfannenstiel or lower midline incisions were preferred for specimen extraction and anastomosis. Stoma site extractions were used when an ostomy was indicated, primarily in cases of obstruction, perforation, or poor distal perfusion. Intracorporeal anastomoses were not routinely performed during the study period.

Age and body mass index (BMI, kg/m²) were recorded as demographic details, and the grade per American Society of Anesthesiologists (ASA) grade (I-III), diabetes status, and immunosuppressant use (e.g., corticosteroids, biologics) were recorded as clinical characteristics. Surgical outcomes included surgical site infections (SSIs defined according to Centers for Disease Control and Prevention (CDC) criteria [6]) and incisional hernias, identified through routine clinical examinations and confirmed by computed tomography (CT) scans during the three-year surveillance period. All imaging assessments were independently reviewed by two radiologists and a surgical reviewer, with discrepancies resolved by consensus. Evaluators were not informed of the study hypothesis. Fascial closure at extraction sites was performed using continuous, slowly absorbable monofilament sutures (PDS), with skin closure using staples or subcuticular sutures at the surgeon’s discretion. Cases with missing or incomplete follow-up data were excluded. Data quality was ensured through double-entry verification. Post-hoc power analysis confirmed an adequate sample size (80% power at α=0.05) to detect intergroup differences.

Statistical analysis

Baseline characteristics were summarized using means for continuous variables (age, BMI) and proportions for categorical variables (ASA grade, diabetes, immunosuppression, SSI, hernia). Comparisons between midline and non-midline groups were performed using continuous variables (independent t-tests for age and BMI) and categorical variables (Chi-square tests for ASA grade distribution, diabetes, immunosuppression, and hernia rates), with Fisher’s exact test applied for expected frequencies <5 (e.g., Pfannenstiel, stoma site hernia rates) [7,8]. Pearson correlation coefficients (r) were used to assess associations between risk factors (SSI, diabetes, immunosuppression) and incisional hernia incidence. Statistical significance was set at p < 0.05. Analyses were conducted using SPSS software, version 27 (IBM Corp., Armonk, NY). A post-hoc power calculation, based on an expected hernia rate difference of 15% (midline: 27% vs. non-midline: 12%) and a sample size of 276 (99 midline, 177 non-midline), confirmed 80% power to detect significant differences at α=0.05 [8]. This ensured adequate statistical robustness for primary comparisons.

## Results

This retrospective study analyzed 276 patients who underwent laparoscopic colorectal cancer resection between March 2019 and March 2022 at a UK District General Hospital, with a minimum of three years of follow-up. Patients were stratified by specimen extraction site into midline (99, 35.9%) and non-midline (177, 64.1%) cohorts, with the latter subdivided into transverse off-midline (116, 42.0%), Pfannenstiel (37, 13.4%), and stoma site (24, 8.7%) groups, as shown in Table 1. Baseline characteristics revealed subtle differences across groups. The mean age was 69.9 years in the midline cohort versus 69.4 years in the non-midline cohort (p=0.0028), with the Pfannenstiel subgroup being the youngest (66.6 years) and the stoma site the oldest (74.5 years) (Table 1). The body mass index (BMI) averaged 28.7 kg/m² in the midline group compared to 29.4 kg/m² in the non-midline group (p<0.001), with Pfannenstiel patients having the highest BMI (29.9 kg/m²) (Table 1).

**Table 1:** Patient demographics and clinical characteristics by extraction site ASA = American Society of Anesthesiologists physical status classification; BMI = Body mass index.

Variable	Midline (n=99)	Transverse Off-Midline (n=116)	Pfannenstiel (n=37)	Stoma Site (n=24)	Non-Midline Total (n=177)
Age (mean years)	69.9	68.8	66.6	74.5	69.4
BMI (kg/m²)	28.7	29.6	29.9	28.5	29.4
ASA I	9 (9.1%)	4 (3.4%)	3 (8.1%)	1 (4.2%)	7 (4.0%)
ASA II	48 (48.5%)	66 (56.9%)	22 (59.5%)	10 (41.7%)	98 (55.4%)
ASA III	42 (42.4%)	46 (39.7%)	14 (37.8%)	13 (54.1%)	72 (40.6%)
Diabetes	10 (10.1%)	8 (6.9%)	3 (8.1%)	3 (12.5%)	14 (8.0%)
Immunosuppression	5 (5.1%)	5 (4.3%)	2 (5.4%)	2 (8.3%)	9 (5.1%)
Surgical Site Infection	36 (36.4%)	29 (25.0%)	5 (13.5%)	6 (25.0%)	42 (23.7%)
Incisional Hernia	27 (27.3%)	17 (14.7%)	1 (2.8%)	3 (12.5%)	21 (11.9%)

The American Society of Anesthesiologists (ASA) grade distribution showed a higher proportion of ASA III patients in the midline 42 (42.4%) and stoma site 13 (54.1%) groups compared to transverse 46 (39.7%) and Pfannenstiel 14 (37.8%) subgroups, with a significant difference between midline and non-midline cohorts (p=0.0324, Table 2). Diabetes prevalence was similar across groups (midline 10 (10.1%) vs. non-midline 14 (8.0%), p=0.732), as was immunosuppressant use (midline 5 (5.1%) vs. non-midline 9 (5.1%), p=1.000) (Tables 1, 2). Surgical site infection (SSI) rates were notably higher in the midline cohort (36, 36.4%) compared to the non-midline (42, 23.7%), with the lowest rate in the Pfannenstiel (5, 13.5%) subgroup (Table 1).

**Table 2:** Statistical comparison between midline and non-midline extraction sites χ² = Chi-square statistic; t = t-statistic; ASA: American Society of Anesthesiologists; df = Degrees of freedom. A p-value <0.05 was considered statistically significant.

Comparison Variable	Subgroup Comparison	Test Statistic (df)	p-value
Incisional Hernia	Midline vs All Non-midline	χ²=9.62 (1)	0.0021
Incisional Hernia	Midline vs Transverse	χ²=4.48 (1)	0.0343
Incisional Hernia	Midline vs Pfannenstiel	Fisher’s exact	0.0008
Incisional Hernia	Midline vs Stoma site	Fisher’s exact	0.1859
Age Difference	Midline vs Non-midline	t=3.05 (274)	0.0028
BMI Difference	Midline vs Non-midline	t=3.85 (274)	<0.001
Diabetes Prevalence	Midline vs Non-midline	χ²=0.12 (1)	0.732
Immunosuppression	Midline vs Non-midline	Fisher’s exact	1.000
ASA Grade Dist.	Midline vs Non-midline	χ²=4.59 (2)	0.0324

Incisional hernia incidence, a primary outcome, was significantly higher in the midline cohort 27 (27.3%) compared to the non-midline cohort 21 (11.9%) (p=0.0021, Chi-square test, Table 2). Among non-midline subgroups, Pfannenstiel one (2.8%) incisions had the lowest hernia rate (p=0.0008, Fisher’s exact test), followed by transverse off-midline 17 (14.7%) (p=0.0343, Chi-square test), while stoma site three (12.5%) extractions showed no significant difference from midline (p=0.1859, Fisher’s exact test) (Table 2).

A strong positive correlation was observed between SSI and incisional hernia incidence (r=0.950, p=0.013, Table 3), indicating a robust association. In contrast, neither diabetes (r=0.296, p=0.628) nor immunosuppressant use (r=0.284, p=0.640) showed a significant correlation with hernia incidence (Table 3). These findings highlight the critical influence of extraction site selection and SSI on postoperative hernia risk.

**Table 3:** Correlation between patient/operative factors and incisional hernia development Pearson correlation coefficients (r) with associated p-values reported. A p-value <0.05 was considered statistically significant.

Risk Factor	Correlation Coefficient (r)	p-value
Surgical Site Infection Rate	0.950	0.013
Diabetes	0.296	0.628
Immunosuppression	0.284	0.640

## Discussion

The results of this study demonstrate a clear association between specimen extraction site and incisional hernia risk in laparoscopic colorectal surgery, with midline incisions conferring a significantly higher risk (27.3%) compared to non-midline sites (11.9%, p=0.0021, Table 2). This finding aligns with prior literature, such as Bosanquet et al.’s meta-analysis, which reported midline incisional hernia rates of 20-30% in colorectal surgery [1]. The notably low hernia rate for Pfannenstiel incisions (2.8%, p=0.0008, Table 2) corroborates Singh et al.’s findings of a 3.5% hernia incidence in a similar cohort, attributed to the anatomical advantages of Pfannenstiel incisions, including reduced tension and minimal muscle disruption [2]. Transverse off-midline incisions (14.7%, p=0.0343, Table 2) also showed a reduced hernia rate compared to midline, consistent with Samia et al.’s report of a 10.2% incidence, suggesting that transverse incisions distribute abdominal wall stress more evenly than midline approaches [3].

The strong correlation between SSI and incisional hernia (r=0.950, p=0.013, Table 3) underscores infection as a critical risk factor, supporting Murray et al.’s finding that SSI increases hernia risk with an odds ratio of 3.2 (95% CI 1.8-5.7) [4]. This association likely results from impaired wound healing due to inflammatory responses and bacterial interference with collagen deposition, as described by Sørensen et al. [5]. The higher SSI rate in the midline cohort (36.4% vs. 23.7% in the non-midline cohort, Table 1) may partly explain the elevated hernia incidence, emphasizing the need for robust perioperative infection control measures, such as prophylactic antibiotics and meticulous wound care, as recommended by Ban et al. [6].

Interestingly, diabetes (p=0.732, Table 2) and immunosuppressant use (p=1.000, Table 2) showed no significant association with hernia incidence in our study, contrasting with Yahchouchy-Chouillard et al., who identified diabetes as a risk factor (odds ratio 2.4, 95% CI 1.3-4.5) [10]. This discrepancy may be due to the relatively low diabetes prevalence (6.9-12.5%, Table 1) or effective glycemic control in our cohort, which could mitigate its impact on wound healing, as suggested by Goodenough et al. [11]. Similarly, the lack of association with immunosuppression may reflect the low prevalence (4.3-8.3%) or heterogeneity in immunosuppressive regimens, warranting further investigation.

The comparable hernia rate between stoma site extractions (12.5%) and midline incisions (p=0.1859, Table 2) suggests that stoma sites, often necessitated by clinical indications like colostomy, do not offer a protective advantage over midline incisions. This finding aligns with Lee et al.’s observation that stoma-related incisions carry a hernia risk similar to that of midline approaches due to fascial weakening [12]. The significant difference in ASA grade distribution (p=0.0324, Table 2) between midline and non-midline cohorts may indicate selection bias, as patients with higher ASA grades (e.g., 54.1% ASA III in stoma site) may have been assigned to midline or stoma site extractions due to perceived surgical complexity, as noted by Pereira et al. [13].

Age and BMI differences, although statistically significant (p<0.001 for most comparisons), had minimal clinical relevance, with mean differences of 1-5 years and 0.7-1.2 kg/m² (Table 1). These findings are consistent with those of Itatsu et al., who reported that age and BMI have a limited direct impact on hernia risk compared to surgical factors, such as incision type [14-17]. However, the higher BMI in non-midline subgroups (e.g., 29.9 kg/m² in Pfannenstiel) suggests that surgeons may select non-midline sites for patients with higher BMI to reduce hernia risk, a strategy supported by DeSouza et al. [15]. It is important to acknowledge that the type of colectomy (right vs. left) and the associated anastomotic technique may influence extraction site selection and thereby impact the observed hernia rates [16]. Right hemicolectomies are frequently extracted through a midline incision due to anatomical proximity and ease of exteriorization, whereas left-sided resections more commonly permit Pfannenstiel or transverse off-midline extractions. This intrinsic distribution could partly account for differences in hernia incidence between groups. Furthermore, the growing adoption of intracorporeal anastomosis, particularly facilitated by robotic platforms, may reduce the need for midline extraction during right hemicolectomy, offering a potential strategy to minimize postoperative hernia risk. Future prospective studies stratifying outcomes by laterality and anastomotic technique are warranted to further delineate these factors.

Study limitations

Its retrospective, single-center design may introduce selection and information bias, as extraction-site choice was influenced by surgeon preference and patient characteristics. Complete blinding of evaluators was not feasible, which may have affected the accuracy of hernia detection. Certain potentially relevant variables, including nutritional status, detailed closure techniques, and surgeon experience, were not consistently documented. The analysis did not account for the type of colectomy or anastomotic technique, which could influence both extraction-site selection and hernia outcomes. Future prospective multicenter studies with standardized methodology are warranted to validate these findings.

## Conclusions

Non-midline extraction sites, particularly the Pfannenstiel approach, are associated with lower rates of incisional hernia compared to midline incisions in laparoscopic colorectal surgery. Surgical site infection remains a key determinant of postoperative hernia risk, underscoring the importance of preventive measures. These findings support preferential use of non-midline extraction sites, where clinically feasible, to optimize long-term patient outcomes. Future prospective and randomized studies are warranted to confirm these observations and further refine extraction site selection strategies.

## References

[1] Bosanquet DC, Ansell J, Abdelrahman T (2015). Systematic review and meta-regression of factors affecting midline incisional hernia rates: analysis of 14,618 patients. PLoS One.

[2] Singh R, Omiccioli A, Hegge S, McKinley C (2008). Does the extraction-site location in laparoscopic colorectal surgery have an impact on incisional hernia rates?. Surg Endosc.

[3] Samia H, Lawrence J, Nobel T, Stein S, Champagne BJ, Delaney CP (2013). Extraction site location and incisional hernias after laparoscopic colorectal surgery: should we be avoiding the midline?. Am J Surg.

[4] Murray BW, Cipher DJ, Pham T, Anthony T (2011). The impact of surgical site infection on the development of incisional hernia and small bowel obstruction in colorectal surgery. Am J Surg.

[5] Sørensen LT, Hemmingsen UB, Kirkeby LT, Kallehave F, Jørgensen LN (2005). Smoking is a risk factor for incisional hernia. Arch Surg.

[6] Ban KA, Minei JP, Laronga C (2017). American College of Surgeons and Surgical Infection Society: Surgical Site Infection Guidelines, 2016 Update. J Am Coll Surg.

[7] Charles JP, Rajendran K, Osborn J (2024). An internal audit on patient safety and quality of services in a rural health training center, Coimbatore. Digit J Clin Med.

[8] Balasundararaj D, Prabhu MS, Thilagarajan S, Rajendran K (2024). Analysis of preanalytical errors in clinical biochemistry laboratory of a tertiary care hospital: a retrospective study. J Clin of Diagn Res.

[9] Natarajan I, Rajendran K, Narayanan S, Shanmugam J (2024). Swachh Bharat Mission Gramin: uptake and challenges in rural Coimbatore. J Family Med Prim Care.

[10] Yahchouchy-Chouillard E, Aura T, Picone O, Etienne JC, Fingerhut A (2003). Incisional hernias. I. Related risk factors. Dig Surg.

[11] Goodenough CJ, Ko TC, Kao LS (2015). Development and validation of a risk stratification score for ventral incisional hernia after abdominal surgery: hernia expectation rates in intra-abdominal surgery (the HERNIA Project). J Am Coll Surg.

[12] Lee L, Abou-Khalil M, Liberman S, Boutros M, Fried GM, Feldman LS (2017). Incidence of incisional hernia in the specimen extraction site for laparoscopic colorectal surgery: systematic review and meta-analysis. Surg Endosc.

[13] Pereira JA, Pera M, Grande L (2013). Incidence of incisional hernia after open and laparoscopic colorectal cancer resection (Article in Spanish). Cir Esp.

[14] Itatsu K, Yokoyama Y, Sugawara G (2014). Incidence of and risk factors for incisional hernia after abdominal surgery. Br J Surg.

[15] DeSouza A, Domajnko B, Park J, Marecik S, Prasad L, Abcarian H (2011). Incisional hernia, midline versus low transverse incision: what is the ideal incision for specimen extraction and hand-assisted laparoscopy?. Surg Endosc.

[16] Fink C, Baumann P, Wente MN (2014). Incisional hernia rate 3 years after midline laparotomy. Br J Surg.

[17] Millbourn D, Cengiz Y, Israelsson LA (2009). Effect of stitch length on wound complications after closure of midline incisions: a randomized controlled trial. Arch Surg.

